# The Effect of the Type of Non-Caged Housing System, Genotype and Age on the Behaviour of Laying Hens

**DOI:** 10.3390/ani10122450

**Published:** 2020-12-21

**Authors:** Zofia Sokołowicz, Magdalena Dykiel, Jadwiga Topczewska, Józefa Krawczyk, Anna Augustyńska-Prejsnar

**Affiliations:** 1 Department of Animal Production and Poultry Products Evaluation, University of Rzeszów, Zelwerowicza Street 4, 35-601 Rzeszów, Poland; zosokolo@ur.edu.pl (Z.S.); augusta@ur.edu.pl (A.A.-P.); 2 Department of Food Production and Safety, Carpathion State College in Krosno, Rynek 1, 38-400 Krosno, Poland; magdalena.dykiel@kpu.krosno.pl; 3 Department of Poultry Breeding, National Research Institute of Animal Production, Krakowska Street 1, 32-083 Balice n. Kraków, Poland; jozefa.krawczyk@izoo.krakow.pl

**Keywords:** alternative housing system, behaviour of laying hens, native breed, commercial hybrid, comfort behaviour, agonistic behaviour

## Abstract

**Simple Summary:**

For several years, a constant decreasing trend has been observed in the number of hens housed in the cage system in favour of non-caged systems, i.e., deep-litter, free-range and organic systems. This study investigated the welfare of laying hens in different non-caged housing systems, namely a deep-litter barn system, a free-range system and an organic system. The study was conducted on hens of a native breed Green-legged Partridge (Z-11) and Hy-Line Brown hybrids. Dustbathing, scratching, wing-leg stretching, wing flapping and preening were recorded as comfort behaviours. Pecking, fighting, threatening and chasing were recorded as agonistic behaviours. The native breed of hen chose to use the outdoor area more often than the commercial breed of hen, which may be a result of better adaptation to the local environmental conditions. The type of non-caged egg production system influenced the percentage of hens displaying comfort and agonistic behaviours in these laying hens. A greater proportion of comfort behaviours were observed in the free-range system and organic system compared with the deep-litter system, which may indicate a higher level of behavioural welfare of laying hens in these systems.

**Abstract:**

This study investigated the welfare of laying hens in different non-caged housing systems, namely a deep-litter barn system (BS), a free-range system (FRS) and an organic system (OS). The study was conducted on 270 hens of a native breed Green-legged Partridge (Z-11) and 270 Hy-Line Brown hybrids. Visual scans were performed to record behaviour of hens. Hens were housed in groups of 30 and observed over the course of one day at 20, 36 and 56 weeks of age. Dustbathing, scratching, wing stretching, wing flapping and preening were recorded as comfort behaviours. Pecking, fighting, threatening and chasing were recorded as agonistic behaviours. The percentage of run use was higher in native hens than in commercial hens (*p* < 0.05). The proportion of hens exhibiting comfort behaviours housed in the FRS and OS was similar but over twice as high as in the BS (*p* < 0.05). In the FRS and OS, the percentage of hens displaying comfort behaviours increased with age (*p* < 0.05). In all the production systems, the percentage of birds displaying comfort behaviours was higher in native breed hens than in commercial breeds (*p* < 0.05). In the BS, the higher proportion of hens displaying an agonistic behaviour was seen more in commercial breed than in the native breed hens (*p* < 0.05). The percentage of birds displaying an agonistic behaviour declined with hen age, both in commercial and native breed hens.

## 1. Introduction

In accordance with [1], table eggs can be produced in cage, deep-litter, free-range and organic systems. For the past several years, a constant decreasing trend has been observed in the number of hens housed in the cage system in favour of non-caged systems, i.e., deep-litter, free-range and organic systems. Although the dynamics of these changes varies between countries, the proportion of hens kept in cage systems in Europe fell by nearly 10% between 2010 and 2015—from 65.5% to 56.1% [2]. In 2017, the number of hens kept in cage systems in the European Union dropped to 53.2%, and in 2019 to 48%. Thus, more and more hens in the European Union are being kept in alternative systems [3]. This trend results from a widespread public opinion that non-caged systems assure better welfare standards for laying hens, especially because they provide birds with freedom to display their natural behaviours [4]. This trend is due not only to the voluntary decision of egg producers, but also to the planned tightening of legislation in this regard. France plans to complete the sale of cage rearing eggs in 2022. Germany plans to ban cage rearing from 2025. This year, the Czech Parliament adopted a ban on the cage rearing of hens from 2027 and called the European Commission to submit proposals to ban the cage rearing of laying hens throughout the EU by 2030. 

As a result of pressure from consumers and animal welfare groups, behaviour welfare has become an important factor of market innovations which has led to diversification of categories of commercially available table eggs, enabling consumers to make informed decisions on purchasing eggs produced in their preferred egg production system [5,6,7]. Consumers can learn about the system in which an egg was produced from the alpha-numeric label on each egg in which the first digit signifies the housing system [8]. Many consumers choose to purchase eggs from a non-caged system despite their higher price [9,10]. 

Diversity of non-caged systems calls for an understanding of the differences between the housing systems in terms of laying hen welfare [7]. Non-caged systems differ in stocking density and quality of housing conditions [11]. According to some authors [12], keeping laying hens in rearing systems with access to open-air outdoor areas compared to other rearing systems provides the greatest potential for improved welfare in terms of behavioural freedom [12]. The behaviour of animals is a key indicator of their welfare status and a source of information on their perception of their housing conditions [13,14,15,16,17]. According to [18], free-range housing systems improve the health and welfare of hens, as it allows the birds to move freely and exhibit natural behaviours in their enclosures. Free-range rearing systems can, however, result in adverse effects on birds as well. Singh et al. [19] report a higher risk of thermal stress in free-range housing systems than in caged systems. While the authors of [20] point to greater health problems in free-range systems, Singh et al. [19] signal a higher level of bird mortality in flocks kept in free-range. Chickens restricted to enclosure are also much more likely to be attacked by predators [21] and parasites [22]. In non-cage systems, there is a higher risk of coccidiosis and a higher incidence of bacterial infections, such as rubella, E. coli, pasteurellosis, histomoniasis and ascariasis in comparison to the cage systems [23].

A large proportion of comfort behavioural patterns and minor appearance of agonistic behaviours can especially testify to a high level of welfare of hens kept in a given production system. Stretching of the wings, wing flapping, dustbathing, sunbathing and preening are often referred to as comfort behaviours [24,25]. Agonistic behaviour is any social behaviour-related fighting. Such behaviour has different forms (threats and intense agonistic behaviour) and is divided into numerous types such as still threats, chasing, aggressive pecking, and attacks [26,27].

The influence of genetics on laying hen welfare is clear, with effects on traits including feather pecking, plumage condition, and associated mortality [28,29,30,31,32] and fearfulness [33,34]. Native breeds are characterised by good immunity and capability of adapting to the environmental conditions [35], while commercial hybrids, for many generations, have been selected for high productivity within controlled environmental conditions [36]. Studies on behaviour and stress in three breeds of laying hens kept in the same environment have shown that compared to Polbar hens and the commercial strain Leghorn, Green-legged partridge hens exhibited the lowest stress levels, which may indicate that their behavioural needs were best met in free-range conditions [37].

The scientific literature includes many results of studies on the impact of a housing system on the welfare of laying hens. However, there are few studies on comparison of the behaviours of native breeds and commercial hybrids in non-caged systems and the impact of hen age on welfare. The aim of the present study was to assess the effect of non-caged housing system (deep-litter, free-range and organic), genotype and age on the welfare of laying hens. It was predicted that in the range systems (FRS and OS), the number (share, percentage) of individual hen behaviours may be different from that in the barn system (BS).

## 2. Materials and Methods

### 2.1. Birds and Management

The research did not require the consent of the ethical committee in accordance with the current National Regulations. The study was conducted in Poland (EU) at the Experimental Station of the National Research Institute of Animal Production. Geographical coordinates: 50°17′13″ N; 21°25′26″ E. The experiment involved 540 hens in total, including 270 hens of the native breed Green-legged Partridge (Z-11) included in a conservation program in Poland and 270 commercial Hy-Line Brown hybrids. The breeding, hatching, and rearing up to the 16th week was carried out at the experimental farm. Hy-Line birds had their beaks trimmed at 10s day old. The beaks were trimmed by employees of a specialist company, using the hot blade method. At 16 weeks of age, 90 hens of Z-11 (3 subgroups of 30 each) and 90 commercial hybrids (3 subgroups of 30 each) were assigned to each of the following housing systems: litter barn (group BS), free-range (group FRS) and organic (group OS), until 56 weeks of age. The hens in each of the studied housing systems (BS, FRS and OS) were kept in a separate building within the same experimental farm. The buildings were the same size; each building had 40 compartments. Each poultry house was divided into compartments. There was a single outdoor area exit from each compartment. Six of the compartments (3 per strain) per house were occupied by the birds on this study. The study compartments were located in the middle of the building, away from the door. Hens were also kept in all other compartments, but no tests were carried out on them other than recording the results of laying and feed consumption.

The birds of the BS group were housed in a poultry house with windows (window area-to-floor area ratio of 1:15) in deep litter without access to a run (paddock). Indoor stocking density was 6 hens/m^2^. Hens from the FRS group were housed in a poultry house with windows (window area-to-floor area ratio of 1:15) in deep litter with free access to a grass-covered open-air run. Indoor stocking density was 6 hens/m^2^, while outdoor stocking density was one laying hen per 4 m^2^. The OS group hens were housed according to regulations pertinent to organic housing, i.e., EC Directive 1804/1999 and Regulation of the European Economic Community (EEC) Council 2092/91. Hens of this group were housed in a poultry house with windows (window area-to-floor area ratio of 1:15) in deep litter (6 hens/m^2^) with free access to a grass-covered open-air run with growing trees, while outdoor stocking density was one laying hen per 5 m^2^. In the FRS and OS, between 6 am and 10 pm, the hens had an unrestricted access to a run through 40 (×) 45 cm openings located on the long wall of the building. The light schedule in the poultry house was the same for all groups and comprised 16 h light and 8 h dark (16L:8D). In autumn and winter, when the natural day was shorter than 16 h, daylight was complemented with artificial light. In each tested housing system, the indoor sheds were equipped with round feeders, drinkers and nests. Feeder diameter was 37 cm; there were 9 nipple drinkers per compartment and 9 single nests per compartment. In the FRS and OS groups, drinkers were also available in the run. Z-11 hens are light-type hens with nutritional requirements similar to Hy-Line hens. Birds of the BS and FRS groups were fed ad libitum with a loose concentrate layer feed (16.08% protein, 11 MJ), and the OS group hens were fed ad libitum with organic poultry feed (16.0% protein, 11 MJ). The layer feeds used in all groups did not contain colour feed additives.

### 2.2. Behaviour

At 20, 36 and 56 weeks of age, laying hens housed in the BS, FRS and OS were observed in order to determine run use frequency and proportion of different forms of comfort and agonistic behaviours. The 20th week of hen age fell in the autumn, the 36th week in winter and the 56th week in spring. During the study period, the average indoor temperature in the poultry house was 18.1 ± 1.8 °C; while in the run, it reached 13.5 ± 2.0 °C at 20 weeks, −2.4 ± 4.1 °C at 36 weeks and 22.7 ± 1.9 °C at 56 weeks of hen age.

Behavioural observations were carried out by 3 observers in the BS and by 6 observers in the FRS and OS, i.e., by 3 observers in the poultry house and 3 observers in the run. Each week of observations (week 20, 36, 56), there were observations in the OS system on Monday, in the FRS system on Tuesday, and in the BS system on Wednesday. All observers participating in the study had been trained during previous pilot tests. Estimation of the level of rater compliance (inter-rater reliability) was verified using Cohen’s kappa coefficient. The obtained compliance rates were above 0.75. After entering the study area, the observers waited for 5 min to allow the birds to become accustomed to their presence, and then they commenced the observations. Four times of the day were chosen to record different forms of behaviour, namely morning (6:00–8:00), late morning (10:00–12:00), afternoon (14:00–16:00) and evening (18:00–20:00), when observations were carried out in all the housing systems. The statistical analysis of the measurements performed at different time points was made using the analysis of variance (ANOVA) method with repeated measurements. At each time of day (morning, late morning (near noon), afternoon, evening) on an evaluation date, each of the nine observed behaviours (dustbathing, scratching, wing-leg stretching, wing flapping, preening, pecking, fighting, threatening, chasing) was scanned three times over the course of 10 min. Each scan lasted 60 s, and within that scan, hens displaying one of the behaviours of interest were counted. 

In total, 9720 scans were taken across all production systems, times of day and hen ages (in the building: 2 genotypes (×) 3 systems (×) 3 subgroups (×) 4 observations period (×) 3 ages (×) 3 replications (×) 9 forms of behaviours = 5832) and in the run (2 genotypes (×) 2 systems (×) 3 subgroups (×) 4 observations periods (×) 3 ages (×) 3 replication (×) 9 forms of behaviours = 3888). The observations covered comfort and agonistic behaviours as presented in Table 1.

### 2.3. Statistical Analysis

The obtained data were collated and submitted for statistical analysis using Statistica 13.3 (StatSoft Polska, Visual Basic, TIBCO Software Inc., Kraków, Poland). The results on the effect of breed on hens using the run, the effect of breed on displaying comfort behaviours and the effect of breed on displaying agonistic behaviours were verified with the use of non-parametric Kruskal–Wallis tests. The proportion of different forms of behaviour were expressed as percentages of birds displaying a specific behaviour. Differences were considered as significant if *p* < 0.05. The data on the effect of genotype, housing system and layer age on the hen welfare were subjected to the multi-factorial analysis of variance, and the main effects (G—genotype effect, S—housing system effect, T—age effect) and an interaction between factors (GxS, GxT, SxT, GxSxT) were determined. The impact of genotype, housing system and age on the hen welfare was analysed with the use of ANOVA, and the following observations were defined for the data analysis: in the building: 2 genotypes (Green-legged Partridge hens, Hy-Line Brown) (×) 3 systems (BS, FRS, OS) (×) 3 subgroup (×) 4 observations period (morning 6:00–8:00, late morning 10:00–12:00, afternoon 14:00–16:00, evening 18:00–20:00) (×) 3 age (20, 36, 56 weeks of age) (×) 3 replications); in the run: (2 genotypes (Green-legged Partridge hens, Hy-Line Brown) (×) 2 systems (FRS, OS) (×) 3 subgroups (×) 4 observations period (morning 6:00–8:00, late morning 10:00–12:00, afternoon 14:00–16:00, evening 18:00–20:00) (×) 3 age (20, 36, 56 weeks of age) (×) 3 replications). In order to evaluate the frequency of hens using the run in each group and subgroup at the analysed times, the result was calculated as a percentage ratio of hens in the run to the number of hens in the group.

## 3. Results 

### 3.1. Run Use

Data on the percentage of Z-11 and Hy-Line Brown hens using the run as presented in Table 2, and on laying hen behaviours at different weeks of age (20, 38, 58), as shown in Table 3 and Table 4, are the mean values based on observations carried out at different times of day (morning, late morning, afternoon and evening). 

The study revealed the effect of the housing system on the percentage of run use (*p* < 0.05). Among hens housed in the free-range system (FRS) and the organic system (OS), a greater proportion of Z-11 hens used the run at all assessment times than among the Hy-Line Brown hens. Hens of both genotypes chose to use the run more frequently at 20 weeks of age and 56 weeks of age than at 36 weeks of age (Table 2). 

### 3.2. Behaviours

**Figure 1:**
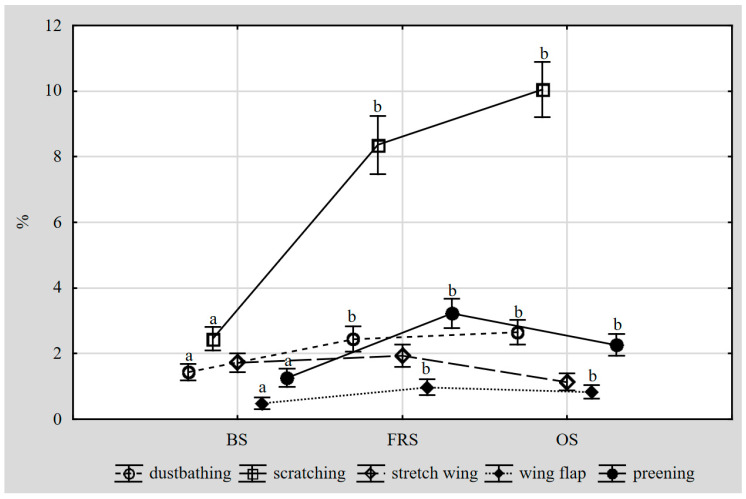
Percentage of different forms of comfort behaviour in the studied housing systems Explanations: housing system: BS—barn deep-litter system, FRS—free-range system, OS—organic system; a, b—values with different letters within the form of behaviour differ significantly at *p* < 0.05.

In Table 3, the percentages of hens expressing dustbathing, scratching, wing-leg stretching, wing flapping and preening behaviours in different housing systems were totalled to give the percentage of comfort behaviour. In Table 4, the percentage of birds displaying a specific behaviour (pecking, fighting, threatening, chasing) in different systems were combined and presented as a proportion of agonistic behaviours. Figure 1 shows the percentage of birds displaying different forms of comfort behaviour in each studied housing system.

The percentage of birds displaying comfort behaviours depended on the housing system, genotype and hen age (Table 3). The proportion of layers engaged in comfort behaviours in the free-range system (FRS) and organic system (OS) was similar (*p* > 0.05), and almost twice as high as in the deep-litter BS (*p* < 0.05). In the FRS and OS, the percentages of birds displaying dustbathing, scratching, wing flapping and preening were similar (*p* > 0.05) and higher than in the BS (*p* > 0.05) (Figure 1). In all housing systems, scratching dominated amongst all the comfort behaviours (Figure 1). In the FRS and OS systems, the proportion of comfort behaviours rose with hen age (*p* < 0.05). In all production systems, the Z-11 hens presented a greater percentage of birds displaying comfort behaviours than the Hy-Line Brown hens (*p* < 0.05).

Independently of bird age, at all assessment times, different forms of agonistic behaviour (pecking, fighting, threatening, chasing) were observed only in the deep-litter system (BS) but no agonistic behavioural patterns were noted in conditions of the free-range (FRS) and organic (OS) systems. A higher percentage (*p* < 0.05) of agonistic behaviours was observed in the Hy-Line Brown hen group behaviour (pecking—0.92%, fighting—0.25%, threatening—1.11%, chasing—0.36%) than in the Z-11 hens (pecking—0.50%, fighting—0.22%, threatening—0.36%, chasing—0.22%). In both the Hy-Line Brown and Z-11 groups, the proportion of agonistic behaviours declined with age (Table 4).

## 4. Discussion

The present study revealed that the percentage of hens using the run in the FRS and OS was high and exceeded 50% in autumn and spring. It is in accordance with the investigations of other authors who demonstrated that birds maintained in small flocks were eager to use grass-covered runs [38,39,40]. Run access is important for laying hen welfare because it allows them to perform a natural behavioural repertoire freely [15,29,41], but it increases the risk of disease, predation and mortality [21]. Run use by laying hens depends on many factors, including the season, atmospheric conditions and botanical composition of vegetation in the run [4], which explains the smaller proportion of hens using the run in winter. In our study, the percentage of hens using the run was reduced during winter, which corresponds with the results by other authors [38,42,43]. Probably, birds reluctantly go to the run on cloudy days when light intensity is higher in the poultry house than outside [13]. Apart from light, the run use by laying hens may also depend on the temperature, which perhaps explains the lower percentage of run use in winter. In addition, this study [13] demonstrated an increased percentage of hens using the run from May till mid-winter. Moreover, in spring, summer and autumn, both in the FRS and OS, the run was covered by grass, so hens could go out to the run to forage for additional food (insects, seeds). 

In our study, the percentage of hens using the run in the studied period was between 60.41 and 64.31%, so never were all birds seen to be in the run at the same time. It is difficult to explain why hens of the same flock, maintained under identical conditions, differ in their interest in the run, and some of them use it every day while others do not go to the run at all. Behavioural observations with the use of sensors revealed that some hens constantly stayed in the house and did not use the run [43,44,45]. They indicated that independently of stocking density (from 2000 to 20,000 hens/ha), ca. 2% of hens did not use the run while the great majority (from 66.5 to 80.5%) used it every day [46]. The access to the run makes the environment more varied, but also increases the hazard caused by predators [47].

In our study, run use depended on the genotype and age of birds, which is in accordance with the data by other authors [29,38,48]. At all observation times, Z-11 hens more willingly used the run than Hy-Line Brown laying hens. The greater interest of the native-bred hens (Green-legged Partridge) in using the run compared with commercial breeds (Hy-Line Brown) can be associated with a better adaptation of native breeds to local, changing and even extreme environmental conditions. The lower interest of the Hy-Line Brown hens in the run can result from a higher level of fear and stress in commercial hens [37]. 

Probably, the hens of commercial breeds, undergoing selective breeding for many generations to improve their performance, use the run more reluctantly because of a higher level of fear and stress to enter the run. Birds of different breeds vary greatly in terms of stress levels, despite the same environmental conditions. The lowest levels of stress indicators were found in the Green-legged Partridge hens, indicating their behavioural needs were best met by that environment [37]. According to [36], long-term selection to improve the productive characteristics of laying hens intended for cage breeding was carried out in stable environmental conditions, in contrast to non-cage systems, where birds live in more variable environmental conditions and could interact in various ways. Results of studies demonstrated that hens preferring to stay in the poultry house showed a higher level of fear, and less efficiently coped with possible environmental stress in the run [45,49,50].

The present study showed that the proportion of different forms of comfort and agonistic behaviour varied between the studied housing systems. The percentage of birds displaying specific forms of comfort behaviour was higher in the FRS and OS than in the BS. Authors investigating the behaviour of hens with brown plumage (Hy-Line Brown, Bovans Brown) and white plumage (DeKalb White and Hy-Line W36) in the deep-litter system showed that such behaviours as perch use, wing flapping and dustbathing required a certain space and depended on the genotype of hens [51]. Agonistic behaviours were observed neither in the free-range system nor the organic system, which can be associated with a beneficial effect of run access on the welfare status of laying hens. Some authors found out that in the hen aviary systems, the hens exhibited more aggressive pecking, but depending on the age, 0.5–0.8% of the hens showed aggressive pecking in the free-range breeding [52,53].

According to results of other studies [21,29,53,54,55,56], vegetation in the run had a beneficial effect on the time spent therein, and could contribute to stress reduction in hens. In our study, this relationship was confirmed since in the housing systems with run access, no agonistic behaviours were recorded. Most likely, a greater area available to a bird and a more enriched environment in the rearing systems with outdoor run access favourably influenced the relationships between the birds. The authors of [56] revealed that social factors, including space allowance per bird, influenced hen behaviour, while [57] documented a relationship between stocking density and manifestations of aggression. In accordance with [14], self-pecking was rare in backyard poultry. On the other hand, it was indicated that environmental enrichment facilitated expression of natural forms of behaviour, including foraging, thus reducing feather pecking [58]. If feather pecking is a kind of behavioural anomaly in hens which can be enhanced by fear and stress [58,59] or behavioural hyperexcitability resulting from neurological changes [60], it can therefore be assumed that stress level in the free-range system and organic system was lower than in the deep-litter system.

Some authors pointed to the impact of the genotype (breed) on aggressive tendency [61,62], which was also confirmed by our study. Namely, a higher percentage of different forms of agonistic behaviour was observed in the commercial genotypes of Hy-Line Brown hens compared with the native breed Green-legged Partridge hens. Frequency of agonistic behaviours decreased with laying hen age, which can be linked with the fact that the hierarchy in the flock is established in the initial period of the laying phase. Some studies have evaluated the impacts of group size and other factors on aggressive behaviours [63]. The effect of the genotype, age and environmental conditions on the frequency of aggressive behaviours was also observed [64].

## 5. Conclusions

The native breed of hen chose to use the run more often than the commercial breed of hen, which probably may be a result of better adaptation to the local environmental conditions. The type of non-caged egg production system influenced the percentage of birds displaying comfort and agonistic behaviours in these laying hens. A greater proportion of comfort behaviours were observed in the free-range system (FRS) and the organic system (OS) compared with the deep-litter system (BS), which may indicate a higher level of behavioural welfare of laying hens in these systems, although larger trials would be needed to test this in commercial-scale flocks.

## Figures and Tables

**Table 1:** Behavioural categories and definitions.

Behaviour	Description of Behaviours
**Comfort behaviour**	
Dustbathing	Lying on side, scratching at pen floor, rubbing head and neck on floor, opening wings.
Scratching	With claws on the ground or the litter.
Wing-leg stretching	Unilateral backward and downward stretching of wing and leg together.
Wing flapping	Bilateral movement of the wings, including wing raising.
Preening	Lifting feathers and cleaning and realigning them with beak.
**Agonistic behaviour**	
Pecking	Violent pecks directed at another hen (receiver), commonly to the head and neck but could also include feet.
Fighting	Two hens aggressively peck each another; often also leaping, wing flapping.
Threatening	Hen stands face to face with an opponent, neck stretched vertically, and neck feathers erected. There is no physical contact.
Chasing	One hen chasing another, with fast running, no vocalisations, no hopping and no wing flapping. The neck feathers may be erected.

**Table 2:** Percentage of hens using the run.

Age of Laying Hens (Weeks)	Housing System ^1^			
	FRS		OS	
	Green-Legged Partridge (Z-11)	Hy-Line Brown	Green-Legged Partridge (Z-11)	Hy-Line Brown
20	^x^ 80.00 ± 11.45 ^a^	^x^ 50.83 ± 6.48 ^b^	^x^ 81.67 ± 9.99 ^a^	^x^ 58.00 ± 5.8 ^c^
36	^y^ 60.83 ± 11.29 ^a^	^y^ 40.83 ± 14.59 ^b^	^y^ 68.58 ± 5.79 ^c^	^y^ 43.42 ± 9.89 ^b^
56	^x^ 78.33 ± 15.8 ^a^	^x^ 51.67 ± 11.07 ^b^	^x^ 79.1 7± 7.69 ^a^	^x^ 55.00 ± 7.36 ^b^
20–56	60.41 ± 18.78 ^a^		64.31 ± 15.71 ^b^	
***p*** **-value**				
G ^2^	<0.001			
S ^3^	<0.001			
T ^4^	<0.001			
GxS	0.615			
GxT	0.225			
SxT	0.376			
GxSxT	0.058			

Explanations: ^1^ housing system: FRS—free-range system, OS—organic system, ^2^ G—effect of genotype, ^3^ S—effect of housing system, ^4^ T—effect of layer age, a, b, c—values in rows with various superscripts differ significantly (*p* < 0.05); x, y—values in columns with various superscripts differ significantly within factors at *p* < 0.05.

**Table 3:** Percentage of hens displaying comfort behaviours *.

Age of Laying Hens (Weeks)	Housing System ^1^					
	BS		FRS		OS	
	Z-11	Hy-Line Brown	Z-11	Hy-Line Brown	Z-11	Hy-Line Brown
20	^x^ 5.00 ^a^ ± 1.69	5.00 ^a^ ± 3.77	^x^ 10.00 ^b^ ± 3.38	^x^ 15.00 ^c^ ± 3.77	^x^ 10.00 ^b^ ± 4.13	^x^ 8.33 ^b^ ± 1.69
36	^y^ 10.00 ^a^ ± 2.39	6.67 ^b^ ± 1.00	^y^ 20.00 ^c^ ± 8.61	^y^ 12.50 ^a^ ± 6.48	^y^ 20.00 ^c^ ± 4.13	^y^ 12.50 ^a^ ± 2.80
56	^y^ 11.67 ^a^ ± 5.06	5.83 ^a^ ± 2.80	^y^ 21.67 ^b^ ± 17.46	^z^ 22.50 ^b^ ± 9.05	^z^ 27.50 ^b,c^ ± 7.69	^z^ 23.33 ^b^ ± 7.16
20–56	7.36 ± 3.97 ^a^		16.94 ± 10.43 ^b^		16.94 ± 8.73 ^b^	
***p*** **-value**						
G ^2^	<0.001					
S ^3^	<0.001					
T ^4^	<0.001					
GxS	<0.001					
GxT	<0.001					
SxT	<0.001					
GxSxT	<0.001					

Explanations: * comfort behaviours: dustbathing, scratching, stretching the wings, wing flapping, preening, ^1^ housing system: BS—barn deep-litter system, FRS—free-range system, OS—organic system, ^2^ G—effect of genotype, ^3^ S—effect of housing system, ^4^ T—effect of layer age, a, b, c—values in rows with various superscripts differ significantly at *p* < 0.05; x, y, z—values in columns with various superscripts differ significantly within factors at *p* < 0.05.

**Table 4:** Percentage of hens displaying agonistic behaviours *.

Age of Laying Hens (Weeks)	Housing System ^1^					
	BS		FRS		OS	
	Z-11	Hy-Line Brown	Z-11	Hy-Line Brown	Z-11	Hy-Line Brown
20	^x^ 2.42 ^a^ ± 2.13	^x^ 3.42 ^a^ ± 2.87	none	None	none	none
36	^y^ 1.25 ^a^ ± 1.95	^xy^ 2.50 ^b^ ± 2.80	none	None	none	none
56	^z^ 0.25 ^a^ ± 0.89	^y^ 2.00 ^b^ ± 2.36	none	None	none	none
20–56	1.97 ± 2.46		None		none	
***p*** **-value**						
G ^2^	<0.001					
S ^3^	<0.001					
T ^4^	<0.001					
GxS	<0.001					
GxT	0.567					
SxT	<0.001					
GxSxT	0.686					

Explanations: *—agonistic behaviours: pecking, fighting, threatening, chasing—in total, ^1^ housing system: BS—barn deep-litter system, FRS—free-range system, OS—organic system, ^2^ G—effect of genotype, ^3^ S—effect of housing system, ^4^ T—effect of laying hen age, a, b—values in rows with various superscripts differ significantly at *p* < 0.05; x, y—values in columns with various superscripts differ significantly within factors at *p* < 0.05.
